# Structure and assembly of the NOT10:11 module of the CCR4-NOT complex

**DOI:** 10.1038/s42003-023-05122-4

**Published:** 2023-07-17

**Authors:** Yevgen Levdansky, Tobias Raisch, Justin C. Deme, Filip Pekovic, Hans Elmlund, Susan M. Lea, Eugene Valkov

**Affiliations:** 1 RNA Biology Laboratory, Center for Cancer Research, National Cancer Institute, Frederick, MD USA; 2 Department of Structural Biochemistry, Max Planck Institute of Molecular Physiology, Dortmund, Germany; 3 Center for Structural Biology, Center for Cancer Research, National Cancer Institute, Frederick, MD USA

**Keywords:** Cryoelectron microscopy, RNA

## Abstract

NOT1, NOT10, and NOT11 form a conserved module in the CCR4-NOT complex, critical for post-transcriptional regulation in eukaryotes, but how this module contributes to the functions of the CCR4-NOT remains poorly understood. Here, we present cryo-EM structures of human and chicken NOT1:NOT10:NOT11 ternary complexes to sub-3 Å resolution, revealing an evolutionarily conserved, flexible structure. Through biochemical dissection studies, which include the *Drosophila* orthologs, we show that the module assembly is hierarchical, with NOT11 binding to NOT10, which then organizes it for binding to NOT1. A short proline-rich motif in NOT11 stabilizes the entire module assembly.

## Abstract (web summary)

Structural and biochemical characterization of the human, chicken, and Drosophila NOT1:NOT10:NOT11 ternary complexes reveal the evolutionarily conserved NOT10:11 module, hierarchical assembly of the complex.

## Introduction

The CCR4-NOT complex has a critical role in regulating mRNA expression in eukaryotic cells, where it catalyzes the shortening of mRNA poly(A) tails (deadenylation) to initiate mRNA turnover^1^. CCR4-NOT also functions in transcript-specific decay and translational repression, with its subunits providing binding sites for RNA-binding proteins and other factors^2,3^. CCR4-NOT exists in all eukaryotes and comprises a conserved core of six subunits, the deadenylases CCR4 and CAF1 (CNOT6/6 L and CNOT7/8 in humans) as well as NOT1-3 and CAF40 (CNOT1-3 and CNOT9 in humans). In *S. pombe* and *S. cerevisiae*, Ccr4-Not has two additional subunits, NOT4 and CAF130. While CNOT4 is only loosely associated with the complex in humans, CAF130 does not exist outside ascomycetes^2^. In addition to the six core subunits of the CCR4-NOT complex, the NOT10 and NOT11 subunits (CNOT10 and CNOT11 in humans) are conserved and stably incorporated in almost all eukaryotes except some unicellular yeasts^4–6^. NOT10 and NOT11 interact with each other and bind to NOT1 to form the NOT10:11 module, which enhances CCR4-NOT deadenylation activity in vitro^7^. In trypanosomes, NOT10 is essential for mRNA degradation and proliferation in the host bloodstream^8^. NOT10 and NOT11 post-transcriptionally downregulate the immune response to HIV infection in primary T cells^9^. Besides NOT3, the NOT10:11 module may provide an additional link of CCR4-NOT to ribosomes^10,11^.

In this study, we provide biochemical and structural insights into the mechanism of assembly, the architecture of the NOT10:11 module, and how this has been preserved in evolutionary terms. We show that the organization of the NOT10:11 module is highly conserved across species, and a short linear motif in NOT11 determines the stability of the entire module.

## Results and discussion

**Fig. 1:**
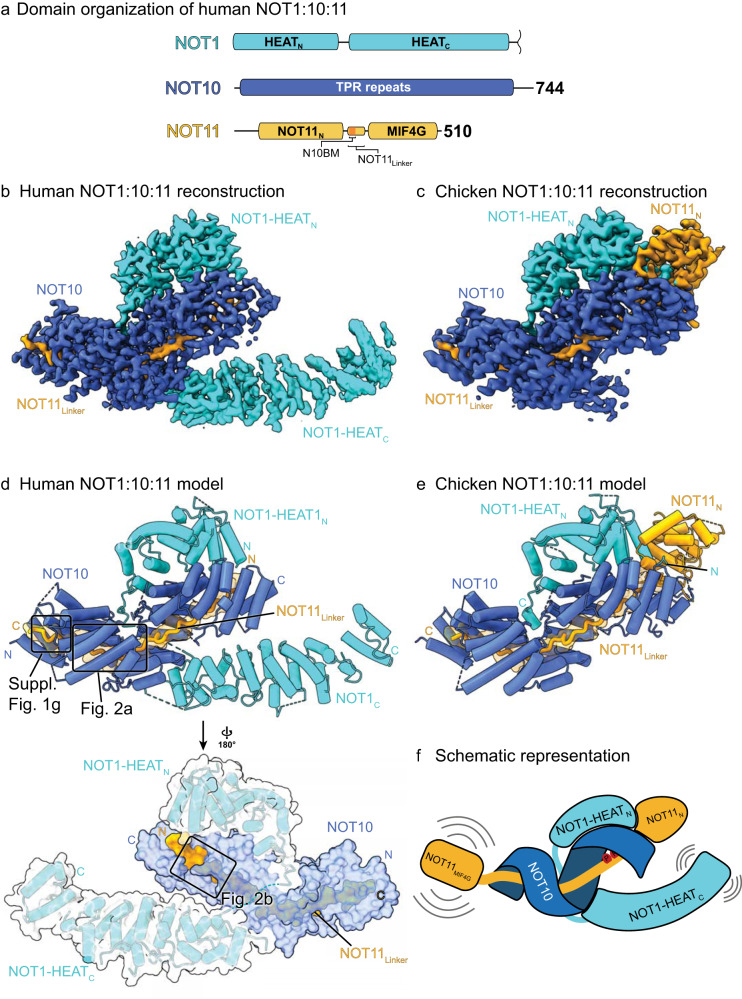
Structure of the vertebrate NOT10:NOT11 module. **a** Domain organization scheme of human NOT10 and NOT11 and the N-terminal portion of NOT1. NOT1 comprises two HEAT repeat domains, HEAT_N_ and HEAT_C_. NOT10 consists of one large TPR repeat domain. NOT11 features an ɑ-helical N-terminal domain NOT11_N_ and a C-terminal MIF4G domain connected by an extended linker, which harbors the NOT10-binding motif NOT10-BM. **b** Cryo-EM reconstruction of the human NOT1:10:11 complex. **c** Cryo-EM reconstruction of the chicken NOT1:10:11 complex. **d** Structure of the human complex. NOT1 comprises two helical repeat domains, and NOT10 is one highly curved TPR repeat domain. Only the extended NOT11_Linker_, bound to NOT10, is ordered, while the NOT11_MIF4G_ domain is flexible and disordered. Boxed regions are shown in more detail in Fig. 2 and Supplementary Fig. 1. **e** Structure of the chicken complex. In contrast to the human complex, the NOT1-HEAT_C_ domain is disordered, but the NOT11_N_ domain is ordered. **f** Schematic representation of the NOT10:11 module. The module assembles around NOT11_Linker_ and NOT10. NOT11_MIF4G_ and NOT1_C_ domains are flexible. A pair of critical prolines in NOT11_Linker_ is marked in red.

The NOT10:11 module is a ternary assembly comprising an N-terminal fragment of NOT1, consisting of two HEAT-repeat domains (HEAT_N_ and HEAT_C_) separated by a flexible linker; NOT10, which is entirely composed of tetratricopeptide (TPR) repeats; and NOT11, which has two distinct domains (NOT11_N_ and NOT11_MIF4G_) separated by a proline-rich linker in most species (Fig. 1a), apart from *Drosophila* NOT11, which lacks the NOT11_N_ but includes the linker.

We reconstituted the ternary complexes of NOT1, NOT10, and NOT11 from chicken (*Gallus gallus, Gg*), fly (*Drosophila melanogaster*, *Dm*), and human (*Hs*) recombinant proteins and determined cryo-EM structures of chicken and human complexes to 2.6 and 2.9 Å resolution, respectively (Fig. 1b–f, Table 1, Supplementary Movies 1, 2).

**Table 1:** Cryo-EM data collection, refinement, and validation statistics.

	Chicken (EMD-29552) (PDB 8FY4)	Human (EMD-29551) (PDB 8FY3)
Data collection and processing	SIMPLE/CryoSPARC/RELION	
Magnification	165,000	
Voltage (kV)	300	
Electron exposure (e–/Å^2^)	51.2 to 58.4	
Defocus range (μm)	−0.25 to –2.5	
Pixel size (Å)	0.723	0.693
Symmetry imposed	C1	C1
Initial particle images (no.)	17,295,060	15,740,546
Final particle images (no.)	760,888	387,942
Map resolution (Å)	2.57	2.88
FSC threshold	0.143	0.143
Refinement	PHENIX/COOT	
Model resolution (Å)	3.0	3.0
FSC Threshold	0.5	0.5
Map sharpening *B* factor (Å^2^)	−98.9	−114.4
Model composition		
Non-hydrogen atoms	7786	8948
Protein residues	988	1123
*B* factors (Å^2^)	106.9	72.5
R.m.s. deviations		
Bond lengths (Å)	0.002	0.003
Bond angles (°)	0.448	0.492
Validation		
MolProbity score	1.48	2.06
Clashscore	5.54	7.8
Poor rotamers (%)	1.75	3.0
Ramachandran plot		
Favored (%)	98.3	96.0
Allowed (%)	1.6	4.0
Disallowed (%)	0.1	0.0

**Fig. 2:**
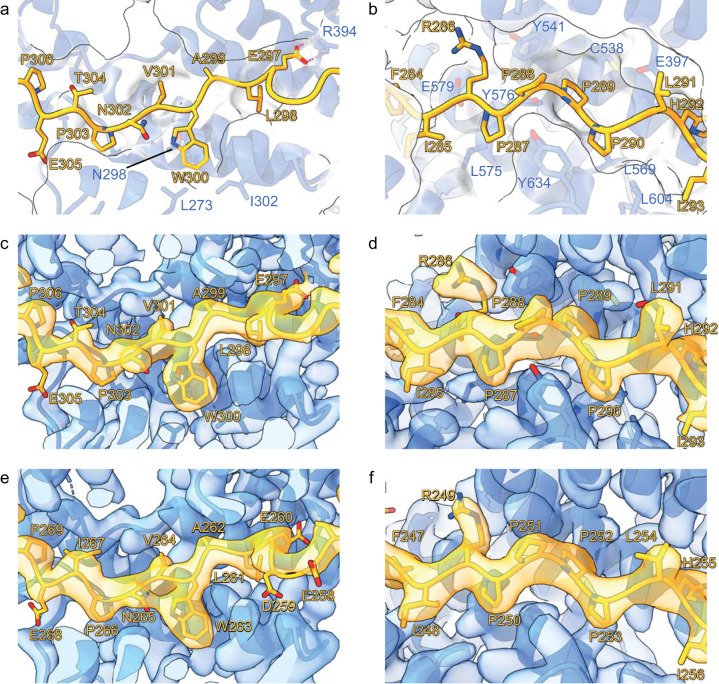
The conserved proline-rich motif in NOT11 stabilizes binding to NOT10. **a** Closeup view of the C-terminal part of the human NOT11_Linker_ bound to NOT10. **b** Closeup view of the human NOT11 proline-rich motif bound to NOT10. **c** CryoEM density for the C-terminal part of the human NOT11_Linker_. **d** CryoEM density for the human NOT11 proline-rich motif. **e** CryoEM density for the C-terminal part of the chicken NOT11_Linker_. **f** CryoEM density for the chicken NOT11 proline-rich motif.

The NOT1 HEAT_C_ domain is disordered in the chicken complex, indicating its flexibility and a peripheral role in supporting module stability (Fig. 1c, e). Still, well-defined density is observed for NOT10, NOT11_N_, NOT11_Linker,_ and HEAT_N_ of NOT1 (Figs. 1b, c and 2a–f, Supplementary Movies 1, 2). HEAT_C_ and HEAT_N_ of NOT1 were resolved in the human complex with NOT10, with only NOT11_Linker_ visible because the N-terminal domain was omitted in the human NOT11 construct (Figs. 1b and 2a–d). The NOT11_MIF4G_ is absent in the density of both structures, consistent with a high degree of flexibility for this domain (Fig. 1f). While this manuscript was in preparation, a crystal structure of the human NOT1:10:11 ternary complex was reported (PDB code 8BFI)^12^ (Supplementary Fig. 1a). A structural alignment of the human and chicken cryoEM structures with this crystal structure, which includes the NOT11_MIF4G_ domain, reveals a very close overall agreement, with only substantial deviation observed for the NOT1 HEAT_C_ domain consistent with its flexibility (Supplementary Fig. 1b). Overall, not only the experimental cryo-EM structures of these ternary complexes agree and superpose well with each other (Supplementary Fig. 1c), there is a striking similarity of the experimental structures to the structures of the human and chicken NOT1:10:11 ternary complexes predicted by AlphaFold2-Multimer^13^ (Supplementary Fig. 1d, e). Encouraged by this, we used AlphaFold2-Multimer to model the flexible NOT11_MIF4G_ domain in the complex and generated a model of the fly ternary complex (Supplementary Fig. 1f).

The overall architecture of human and chicken complexes reveals a heterodimer formed by NOT10 TPR repeats superhelically wrapping around the extended NOT11_Linker_ (Figs. 1d–f and 2). This strikingly extensive NOT10–NOT11_Linker_ interface is predominantly stabilized via conserved hydrophobic contacts. In the human complex, the stacked TPR repeats of the N-terminal portion of NOT10 anchor the C-terminal end of NOT11_Linker_ with the highly conserved L50^NOT10^ and N66^NOT10^ enclosing the conserved aromatic, W312^NOT11^. The pocket is capped by a conserved F38^NOT10^ (Supplementary Fig. 1g). The invariant W300^NOT11^ is contacted by the aliphatic portions of the conserved triad of L273/N298/I302^NOT10^ (Fig. 2a), and here the NOT11_Linker_ principally interacts with the C-terminal portion of NOT10. In addition to its interactions with the N-terminal segment of NOT11_Linker_, the C-terminal half of NOT10 docks on NOT1 HEAT_N_ in both complexes in an identical orientation stabilized via conserved hydrophobic contacts (Supplementary Fig. 1h). Hydrogen bonding between the invariant E297^NOT11^ and R394^NOT10^ initiates the second interacting proline-rich N-terminal segment of NOT11_Linker_ where the highly constrained backbone of the almost invariant P289–P290^NOT11^ pair is oriented around conserved aromatics Y541^NOT10^ and Y576^NOT10^ (Fig. 2b). The invariant aromatic, F284^NOT11^ in human, caps the hydrophobic interactions of NOT11_Linker_. Collectively, this NOT11_Linker_ N-terminal segment effectively organizes the C-terminal NOT10 TPR repeats, which mediate the NOT1-binding site on the opposite interface to NOT11. This observation prompted us to investigate the function of the NOT11_Linker_ in supporting the module stability in vitro.

**Fig. 3:**
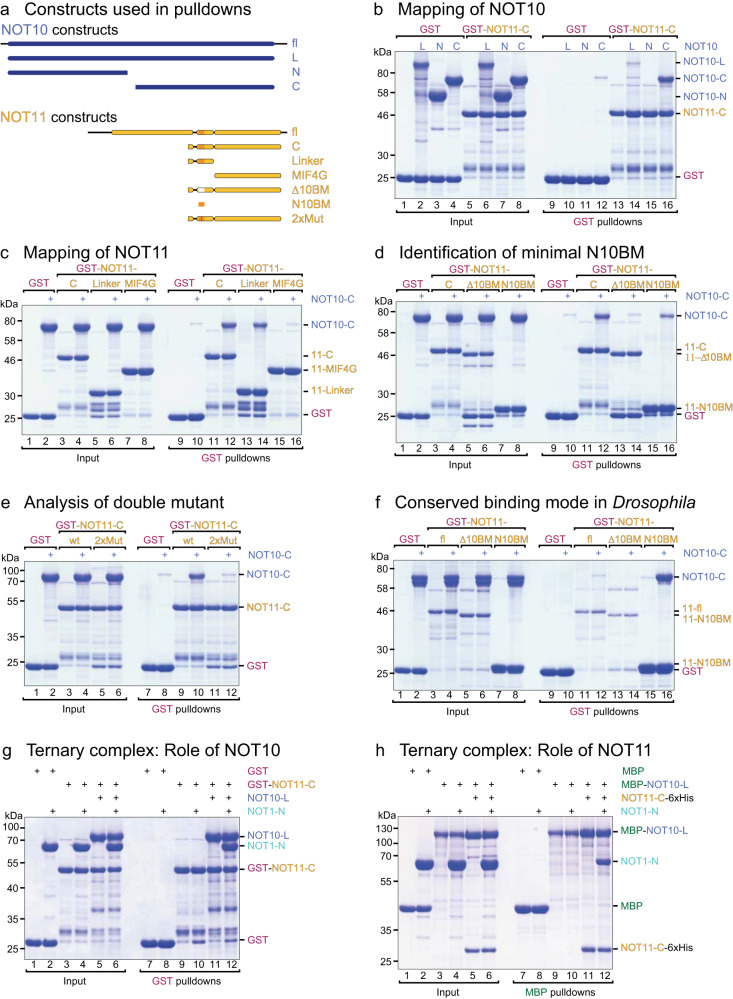
The NOT10:NOT11 module assembles sequentially. **a** Schematic representation of the constructs used in this study. **b** GST pulldown assays to identify the NOT11-binding region in NOT10. **c** GST pulldown assay to identify the NOT10-binding region in NOT11. **d** GST pulldown assay showing that the NOT11 NOT10-binding motif (N10BM) is necessary and sufficient for binding NOT10. **e** A NOT11 P289A–P290A double mutant disrupts NOT10 binding. **f** The NOT10-BM is also necessary and sufficient for the NOT10:NOT11 interaction in *Drosophila*. **g** Unlike the NOT10:NOT11 heterodimer, NOT11 alone cannot associate with NOT1. **h** NOT10 alone cannot bind NOT1, but requires association with NOT11. Source data displaying uncropped gel images corresponding to **b**–**h** are available in Supplementary Figs. 7 and 8.

In pulldown assays with recombinant proteins, we observed not only that a NOT11 construct lacking the NOT11_N_ domain interacted with NOT10 as expected but also that this interaction is supported solely by the C-terminal portion of NOT10 (Fig. 3a, b). Consistent with the structural information (Figs. 1b, c and 2), the interaction is mediated through NOT11_Linker_, while NOT11_MIF4G_ and NOT11_N_ are dispensable (Fig. 3c).

We then sought to dissect the contribution of the extensive NOT11_Linker_ through a series of pulldowns with the truncations of the NOT11 D257–N320 region. Remarkably, a 15-residue motif (E283–E297), termed the NOT10-binding motif or NOT10-BM, was necessary and sufficient to bind NOT10 stably (Fig. 3d, Supplementary Fig. 2). This observation is consistent with previous findings indicating that two NOT11 fragments comprising either M61–P282 or residues E295–K510 failed to interact with endogenous NOT10 in human cell lysates^6^.

Substitution of the highly conserved P289^NOT11^–P290^NOT11^ pair, which contributes to the organization of the C-terminal NOT10 TPR repeats (Figs. 1f and 2b), by alanines (‘2xMut’) abolished the interaction with NOT10 (Fig. 3e). The conservation of the NOT10-BM sequence, and structural comparison suggests that this mode of NOT10 recognition by NOT11, critical to module assembly, is an evolutionarily conserved mechanism (Supplementary Fig. 1f). To test this hypothesis, we observed that *Dm* NOT11 interacts with the NOT10 C-terminal region via an analogous NOT10-BM, which marks the very N-terminus of the *Dm* NOT11 protein (Fig. 3f). This interaction is conserved not only in vertebrates and flies but likely also in plants and fungi (Supplementary Fig. 2).

Short linear motifs are typical for transient and reversible interactions such as the ones recruiting the CCR4-NOT complex to its various mRNA targets^2,14–16^. Our finding that a short motif of NOT11 is necessary and sufficient for stable association with NOT10 in vitro was unexpected as the NOT10:11 module is thought to be a stable, integral part of the CCR4-NOT complex^4–6^. This association of NOT11 with NOT10 is reminiscent of the architecture of the C-terminal NOT1:2:3 module of CCR4-NOT, where the N-terminal regions of NOT2 and NOT3 are critical for stability^17^.

Earlier studies showed a direct interaction between NOT11 and the NOT1 N-terminal region in human^4^ and fly paralogs^6^, suggesting a model where all three proteins interact with each other^4^. We could not reproduce the reported NOT1–NOT11 interaction^4^ using purified proteins, but a NOT10:NOT11 heterodimer efficiently pulled down NOT1 (Fig. 3g), suggesting that NOT10:NOT11 heterodimerization is required for both proteins to bind NOT1. NOT10 contacts NOT1 in human and chicken structures, and the interfacing residues are mostly conserved. However, in protein-protein interaction assays, isolated NOT10 does not bind NOT1; instead, a stoichiometric interaction of NOT1 requires the assembled NOT10:NOT11 heterodimer (Fig. 3h).

We conclude that NOT11 interaction with NOT10 is critical for the heterodimer to dock on NOT1, which scaffolds the CCR4-NOT complex stably. The rigid proline-rich motif in NOT11 likely organizes the locally flexible TPR solenoid of NOT10 to form the NOT1-binding interface. This effectively endows a linear peptide motif to act as a molecular determinant of the entire module assembly (Fig. 1f). Our structural and biochemical dissection of the sequential assembly mechanism of a conserved module of the CCR4-NOT mRNA regulatory complex will facilitate the development of chemical tools to interrogate its role in immunity, development, proliferation, and discovery of new functions.

## Methods

### DNA constructs

For GST pulldowns, cDNAs encoding human (*Hs*) NOT11-C (residues D257–D498) and *Drosophila* (*Dm*) NOT11 (residues M1–E214) were inserted in the pnEA-pG plasmid^18^, resulting in fusion proteins carrying N-terminal GST (glutathione S-transferase) tags cleavable by the HRV3C protease. *Hs* NOT11-C (residues D257–D498) cDNA was inserted into the pnEA/vH plasmid^18^ in-frame with a C-terminal, hexahistidine (6xHis) tag to be used in MBP pulldowns. A multi-sequence alignment of NOT11 can be found in Supplementary Fig. 2.

cDNA fragments of *Hs* NOT10-L (residues D25–Q707), NOT10-N (residues D25–S331) and NOT10-C (residues T351–Q707) were inserted in the pnYC-pM plasmid^18^ resulting in fusion proteins carrying N-terminal HRV3C-cleavable MBP (maltose-binding protein) tags. *Dm* NOT10-C (residues S337–S635) was inserted in the same vector. A multi-sequence alignment of NOT10 can be found in Supplementary Fig. 3.

For the co-expression with GST-tagged NOT11, the *Hs* NOT10-L (residues D25–Q707) construct was inserted into the pnYC-vM plasmid^18^ in-frame with an N-terminal MBP tag cleavable by TEV protease.

The *Hs* NOT1-N (residues D4–N682) cDNA fragment was inserted into the pnYC-pM plasmid.

Constructs of *Hs* NOT1 (residues M1–D1000) and bicistronic *Hs* NOT10 (residues D25–Q707):NOT11 (residues D257–D498) used for the cryo-EM experiments were described previously^19^.

For expression of the chicken (*Gg)* NOT1:NOT10:NOT11 complex, two plasmids were generated. A cDNA fragment encoding the *Gg* NOT1 N-terminus (residues M1–N682), which does not encode a solubility tag, was inserted into the pnYC vector. cDNA fragments encoding *Gg* NOT10 (residues M24–Q707) and *Gg* NOT11 (residues R23–T460) were cloned in a bicistronic plasmid based on the pnEA backbone, thus resulting in the expression of untagged NOT10 and NOT11 with a C-terminal, TEV-cleavable 6xHis tag.

### Production and purification of recombinant proteins for pulldown studies

All recombinant proteins were produced in *Escherichia coli* BL21 (DE3) Star cells (Thermo Fisher Scientific). Expression was induced overnight in Luria-Bertani (LB) or ZY autoinduction medium at 20 °C. The *Hs* NOT11 and *Dm* NOT11 constructs were produced as fusion proteins carrying N-terminal, HRV3C-cleavable GST tags. The cells were lysed in a buffer containing 50 mM HEPES/NaOH pH 7.5, 300 mM NaCl, 2 mM DTT, supplemented with complete EDTA-free protease inhibitors (Roche), 5 µg/mL DNaseI and 1 mg/mL lysozyme. Centrifugation at 40,000 g for 45 min clarified insoluble cell debris in the lysates. The proteins were isolated from cleared lysates using Protino glutathione agarose 4B beads (Macherey-Nagel) and eluted with the same buffer supplemented with 25 mM reduced glutathione. For GST pulldown assays, the proteins were further purified by anion exchange chromatography using a HiTrap Q column (Cytiva) followed by size exclusion chromatography on a Superdex 200 26/600 column (Cytiva) in a buffer containing 10 mM HEPES/NaOH pH 7.5, 200 mM NaCl, 2 mM DTT.

*Hs* NOT10 and *Dm* NOT10 were produced with N-terminal MBP tags. After lysis in the same buffer as above and supplemented with DNaseI, lysozyme, and protease inhibitors, the proteins were isolated from the cleared lysates using amylose resin (New England Biolabs) and eluted with a buffer supplemented with 25 mM D-(+)-maltose. The eluted proteins were purified by anion exchange chromatography over HiTrap Q columns and size exclusion chromatography Superdex 200 26/600 column.

The NOT10:NOT11 complexes used for interaction studies with NOT1-N were obtained by co-expression of either GST-NOT11-C with MBP-(TEV)-NOT10-L or NOT11-L-6xHis with MBP-NOT10. The NOT10:GST-NOT11 complex was isolated from the lysate using glutathione agarose, as described above. The MBP tag was removed by overnight cleavage using TEV protease, followed by binding the complex to a HiTrap Heparin column (Cytiva) and elution with a gradient of 100–1000 mM NaCl. The complex was applied to size exclusion chromatography on a Superdex 200 16/600 column (Cytiva). The MBP-NOT10:NOT11 complex was isolated from lysate as described above using amylose resin and further purified over HiTrap IMAC (Cytiva), HiTrap Q, and Superdex 200 16/600 columns.

Following purification, proteins, and complexes were concentrated using Amicon centrifugal filter units (Millipore Sigma) to 5–10 mg/ml, flash-frozen in liquid nitrogen, and stored at −80 °C.

### Production and purification of ternary complexes for cryo-EM

Production and purification procedures for the reconstitution of human (*Hs*) and chicken (*Gg*) NOT1:NO10:NOT11 ternary complexes were identical.

*Hs* NOT1 (residues M1–D1000):NOT10 (residues D25–Q707):NOT11 (residues D257–D498) or *Gg* NOT1 (residues M1–N682):NOT10 (residues M24–Q707):NOT11 (residues R23–T460) ternary complexes were co-expressed in *E.coli* BL21(DE3) Star cells (Thermo Fisher Scientific) in LB medium at 20 °C overnight. Harvested cells were resuspended in a buffer containing 50 mM HEPES/NaOH pH 7.5, 300 mM NaCl, and 25 mM imidazole and lysed by sonication. The lysate was clarified by centrifugation at 40,000 *g* for 45 minutes. The proteins were captured from the cleared lysate by loading on a nickel-charged HiTrap IMAC column (Cytiva) and eluted with a linear gradient in the same buffer supplemented with 500 mM imidazole. The collected fractions were diluted at a 1:1 ratio with the buffer containing 50 mM HEPES/NaOH pH 7.5, 100 mM NaCl, and 2 mM DTT, and loaded on a 5 mL HiTrap heparin column (Cytiva). The bound complex was washed with 30 mL of buffer containing 50 mM HEPES/NaOH pH 7.5, 200 mM NaCl, and 2 mM DTT, and subsequently eluted with the linear gradient in the same buffer containing 1 M NaCl. The complex was then loaded and eluted on Superdex 200 26/600 column (Cytiva) equilibrated in a buffer containing 10 mM HEPES/NaOH pH 7.5, 200 mM NaCl, and 2 mM DTT. Peak fractions containing the chicken complex were collected and concentrated using Amicon centrifugal filter units (Millipore Sigma) to 5–10 mg/ml, flash-frozen in liquid nitrogen, and stored at −80 °C. The peak fraction of the eluted human complex, at 1.3 mg/ml, was collected and stored on ice until further use.

For cryo-EM grid preparation, chicken and human complexes were diluted using the buffer from the last purification step. *Hs* NOT1:NOT10:NOT11 grids were prepared at 1 mg/ml with 0.2% w/v CHAPS (Millipore Sigma) or at 0.4 mg/ml without. *Gg* NOT1:NOT10:NOT11 grids were prepared at 0.4 mg/ml with or without DDM (Millipore Sigma) addition (0.005% w/v) or 0.75 mg/ml without DDM. Samples were adsorbed onto glow-discharged Quantifoil-Au 300 R1.2/1.3 or R2/1 holey carbon grids for 10 s and then blotted for 3 s at 10 °C, 100% humidity. Grids were vitrified in liquid ethane using a Vitrobot Mark IV (Thermo Fisher Scientific).

Data were collected using the software EPU (Thermo Fisher Scientific) in counted mode in electron-event representation (EER) format^20^ on a CFEG-equipped Titan Krios G4 (Thermo Fisher Scientific) operating at 300 kV with a Selectris imaging filter (Thermo Fisher Scientific) with a slit width of 10 eV and Falcon 4 direct detection camera (Thermo Fisher Scientific) at ×165,000 magnification (Table 1). Movies were collected at an exposure rate of 12.9–14.7 e^−^/Å^2^/s with a constant exposure time of 3.98 s, resulting in a total exposure of 51.2–58.4 e^−^/Å^2^. Data were collected at tilts up to ±20 degrees after initial analysis, indicating a preferred orientation problem for both samples. Motion correction, CTF estimation, particle picking, particle extraction, and 2D classification of particles were performed in real-time using SIMPLE v3.0.0^21^. For each complex initial selections of particles were made based on the selection of 2D classes from the streaming processing and the various samples (±detergent, ±tilting) and were combined. Ab initio models were generated in CryoSPARC v3.31^22^. 2D classification/selection and regeneration of multiple ab initio classes in CryoSPARC generated the final particle sets polished in RELION v3.1.2^23^, after which final non-uniform 3D refinement^24^ generated the volumes described in Table 1. Volumes were post-processed with DeepEMhancer v0.13^25^ to generate volumes for model building. Details about data acquisition and processing can be found in Supplementary Figs. 4 and 5 and in Table 1.

AlphaFold^13,26^ models were used as a starting point for model building and manually adjusted in COOT^27^. The progress of model building was monitored by real-space refinement in PHENIX^28^ against respective maps. MolProbity^29^ and PHENIX^30^ model validation tools were used to assess the stereochemistry and the correlation between the density maps and the models. Model quality statistics are reported in Table 1. Map-to-model correlation plots are reported in Supplementary Fig. 6.

Figures were created using UCSF ChimeraX^31^ and Adobe Illustrator.

### Reporting summary

Further information on research design is available in the Nature Portfolio Reporting Summary linked to this article.

## Supplementary information

Peer Review File

Supplemental Material

Description of Additional Supplementary Files

Supplementary Movie 1

Supplementary Movie 2

Reporting Summary

## Data Availability

The cryo-EM maps and atomic coordinates for human and chicken NOT1:NOT10:NOT11 complexes are deposited in the Electron Microscopy Data Bank and PDB under the following accession codes: EMD-29551/8FY3 (human) and EMD-29552/8FY4 (chicken). Plasmids created and used in this study will be made available upon request. Source data displaying uncropped gel images corresponding to Fig. 3b–h are available in Supplementary Figs. 7 and 8. Any remaining information can be obtained from the corresponding authors upon request.
