# Supplementary material for: Structure and assembly of the NOT10:11 module of the CCR4-NOT complex
Source: Commun Biol. 2023 Jul 17;6:739. doi: 10.1038/s42003-023-05122-4 (PMC10352241; doi:10.1038/s42003-023-05122-4)
Supplement: Supplementary file 2 — Supplemental Material [file 42003_2023_5122_MOESM2_ESM.pdf]

SUPPLEMENTARY INFORMATION

Structure and assembly of the NOT10:11 module of the CCR4-NOT complex

Yevgen Levdansky, Tobias Raisch, Justin C. Deme, Filip Pekovic, Hans Elmlund, Susan M. Lea & Eugene Valkov

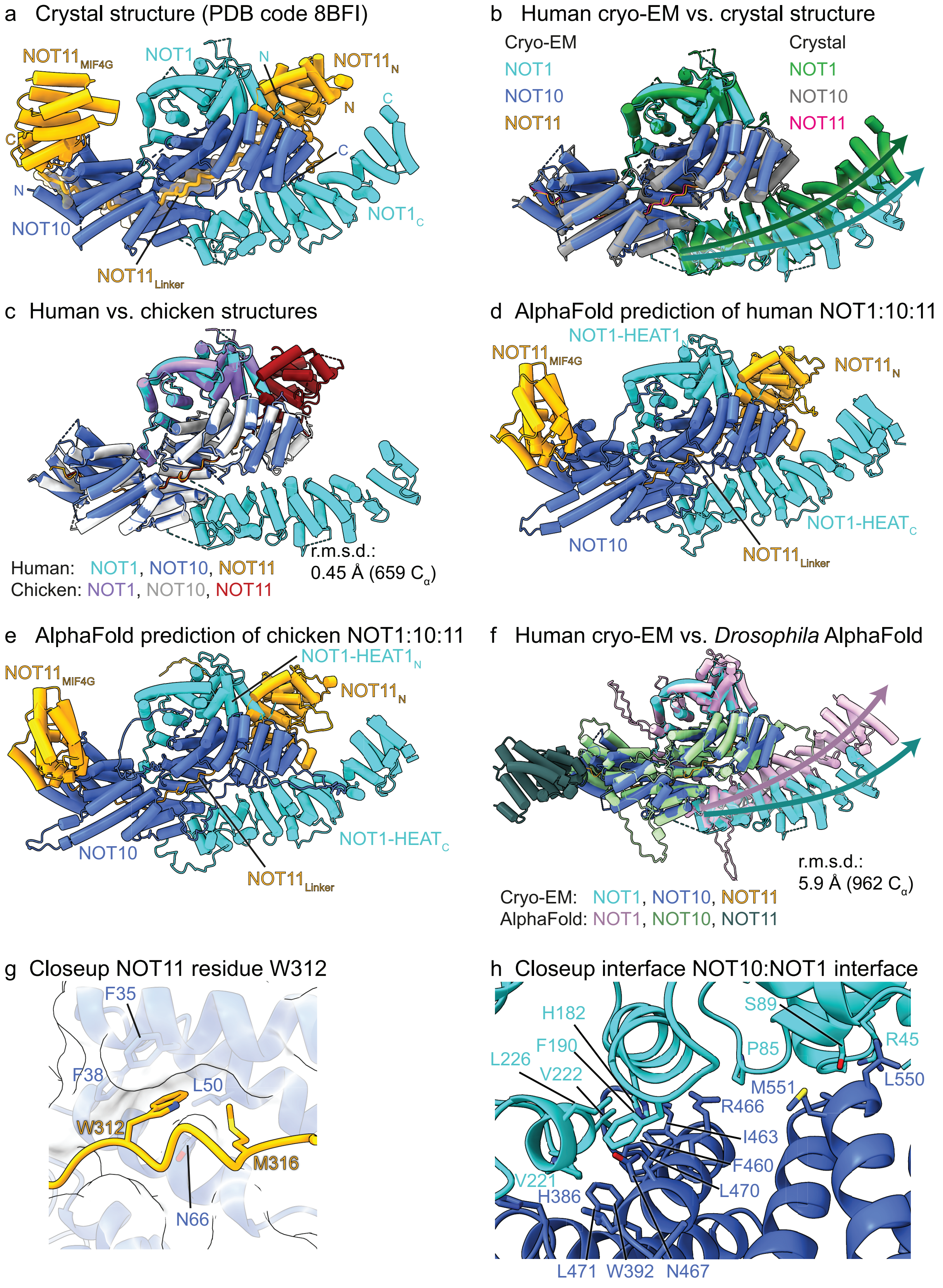
a Crystal structure (PDB code 8BFI)
NOT11MIF4G
NOT1
N
NOT11N
N
C
C
C
N
NOT10
NOT1C
NOT11Linker
b Human cryo-EM vs. crystal structure
Cryo-EM
NOT1
NOT10
NOT11
Crystal
NOT1
NOT10
NOT11
c Human vs. chicken structures
Human: NOT1, NOT10, NOT11
Chicken: NOT1, NOT10, NOT11
r.m.s.d.:
0.45 Å (659 Cα)
d AlphaFold prediction of human NOT1:10:11
NOT1-HEAT1N
NOT11MIF4G
NOT11N
NOT1-HEATC
NOT10
NOT11Linker
e AlphaFold prediction of chicken NOT1:10:11
NOT1-HEAT1N
NOT11MIF4G
NOT11N
NOT1-HEATC
NOT10
NOT11Linker
f Human cryo-EM vs. Drosophila AlphaFold
r.m.s.d.:
5.9 Å (962 Cα)
Cryo-EM: NOT1, NOT10, NOT11
AlphaFold: NOT1, NOT10, NOT11
g Closeup NOT11 residue W312
F35
F38
L50
W312
M316
N66
h Closeup interface NOT10:NOT1 interface
H182
S89
F190
P85
R45
L226
V222
M551
L550
R466
I463
F460
V221
L470
H386
L471
W392
N467

Supplementary Figure 1. Conserved architecture of the NOT1:10:11 complex across species and experimental methods

a) Crystal structure of the human NOT1:10:11 complex.

b) Superposition of crystal and cryo-EM structures of the human NOT1:10:11 complexes.

c) Superposition of chicken and human cryo-EM structures determined in this study.

d) AlphaFold2-Multimer structure prediction of the human NOT1:10:11 complex, including the $NOT11_N$ and domain omitted from the construct used for cryo-EM studies, and the $NOT11_{MIF4G}$, which was disordered in the cryo-EM reconstruction.

e) AlphaFold2-Multimer structure prediction of the chicken NOT1:10:11 complex, including the NOT1-$HEAT_C$ and the $NOT11_{MIF4G,}$ which were disordered in the cryo-EM reconstruction.

f) Superposition of the cryo-EM structure of the human NOT1:10:11 complex with the AlphaFold2-Multimer prediction of the fly complex.

g) Closeup view of $NOT11^{W312}$ binding into a pocket on the N-terminal part of NOT10.

h) Closeup view of the interface between NOT10 (blue) and $NOT1_N$ (cyan). Side chains important for the interface are depicted as sticks.

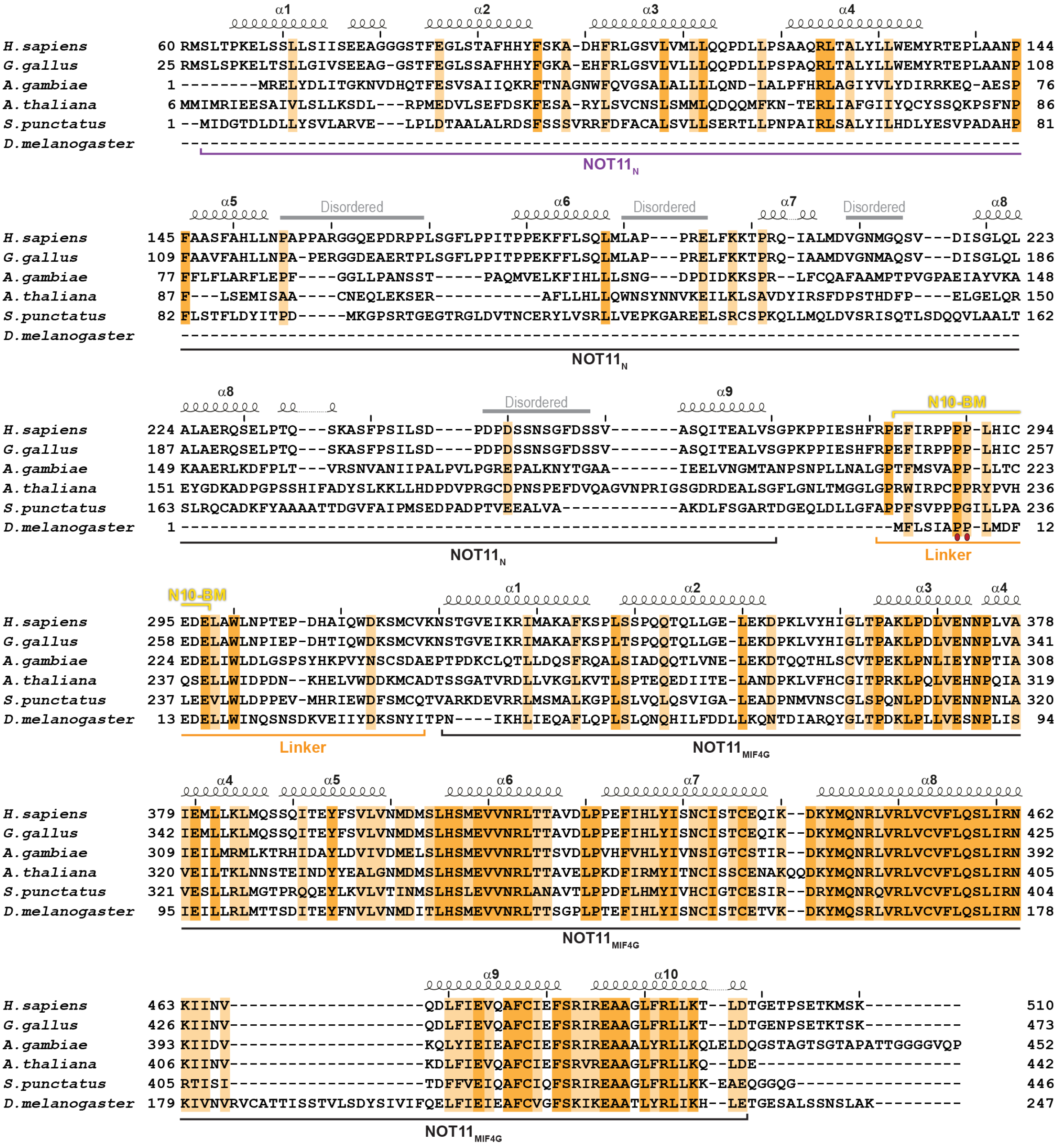

Supplementary Figure 2. Sequence alignment of NOT11.

Alignment using the following eukaryotic species: human (*Homo sapiens*), chicken (*Gallus gallus*), mosquito (*Anopheles gambiae*), thale grass (*Arabidopsis thaliana*), the fungus *Spizellomyces punctatus* and fruit fly (*Drosophila melanogaster*). The NOT10-binding motif (NOT10BM) is indicated above the alignment, whereas domains, the interdomain linker, and the mutated proline residues are indicated below the alignment. Secondary structure elements are shown above the alignment as observed in the chicken structure (in the case of NOT11$_{N}$) and the AlphaFold2 prediction (NOT11$_{MIF4G}$). Residues entirely or highly conserved are highlighted in intense and light orange, respectively.

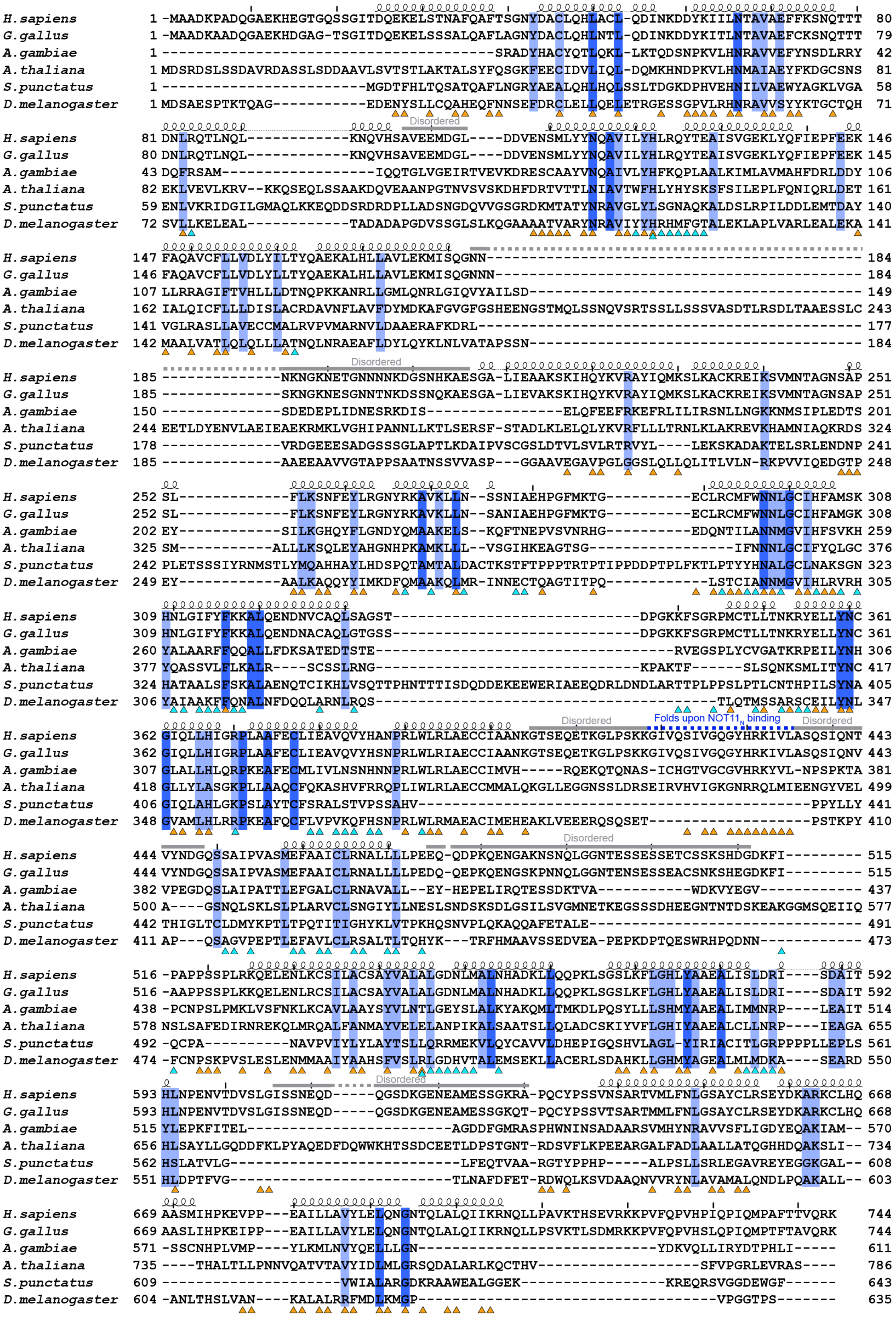

Supplementary Figure 3. Sequence alignment of NOT10.

Sequence alignment of NOT10 of the same eukaryotic species as in Supplementary Fig. 2. Secondary structure is shown above the alignment as observed in the cryo-EM structures. Residues entirely or highly conserved are highlighted in intense and light blue, respectively. Orange and cyan triangles mark residues contacting NOT11 and NOT1, respectively.

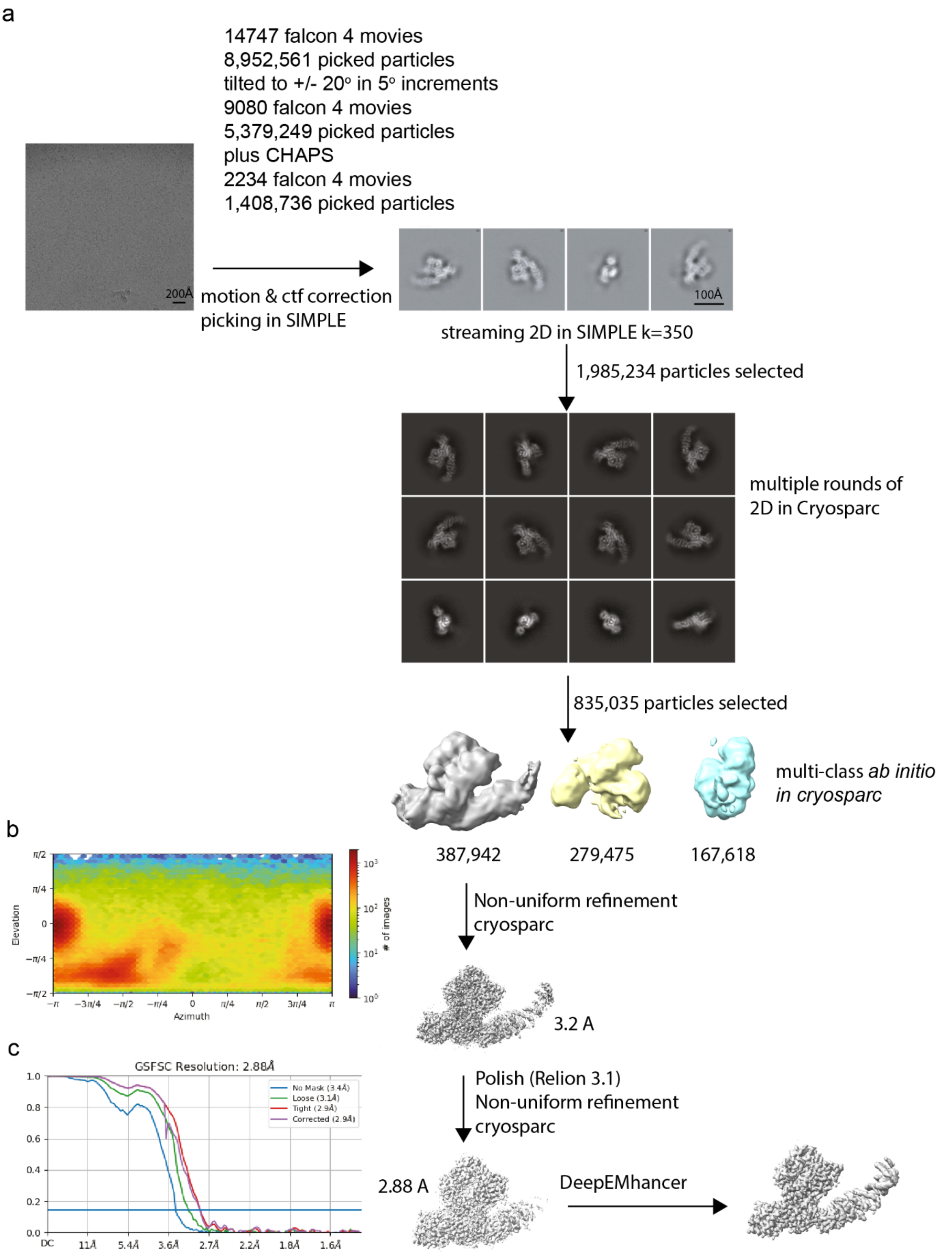

Supplementary Figure 4. Cryo-EM processing workflow of human NOT1:NOT10:NOT11.

a) Image processing workflow, including a representative micrograph.

b) Angular distribution plot.

c) Gold-standard Fourier Shell Correlation (GSFSC) curve.

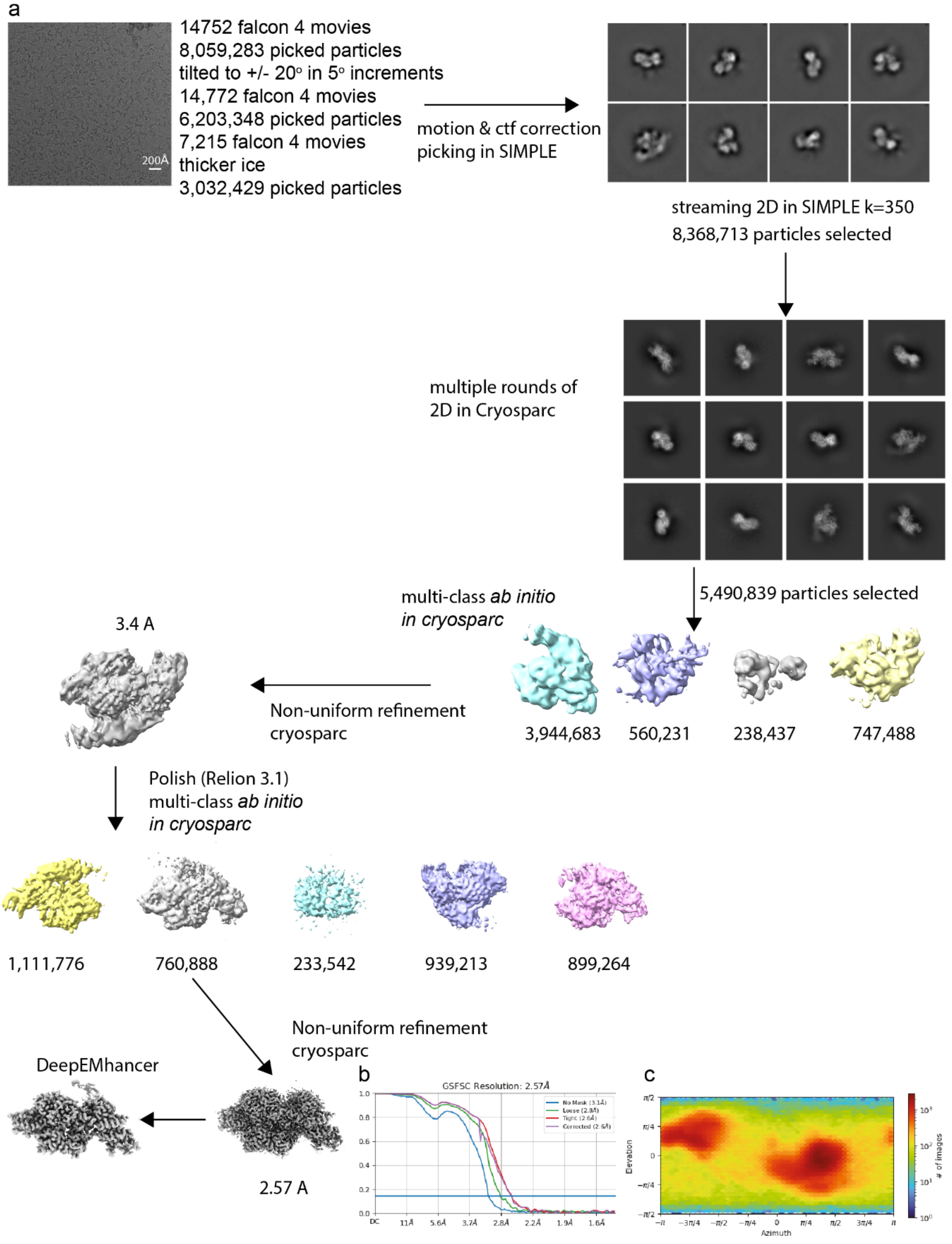

Supplementary Figure 5. Cryo-EM processing workflow of chicken NOT1:NOT10:NOT11.

a) Image processing workflow, including a representative micrograph. **b)** Gold-standard Fourier Shell Correlation (GSFSC) curve. **c)** Angular distribution plot.

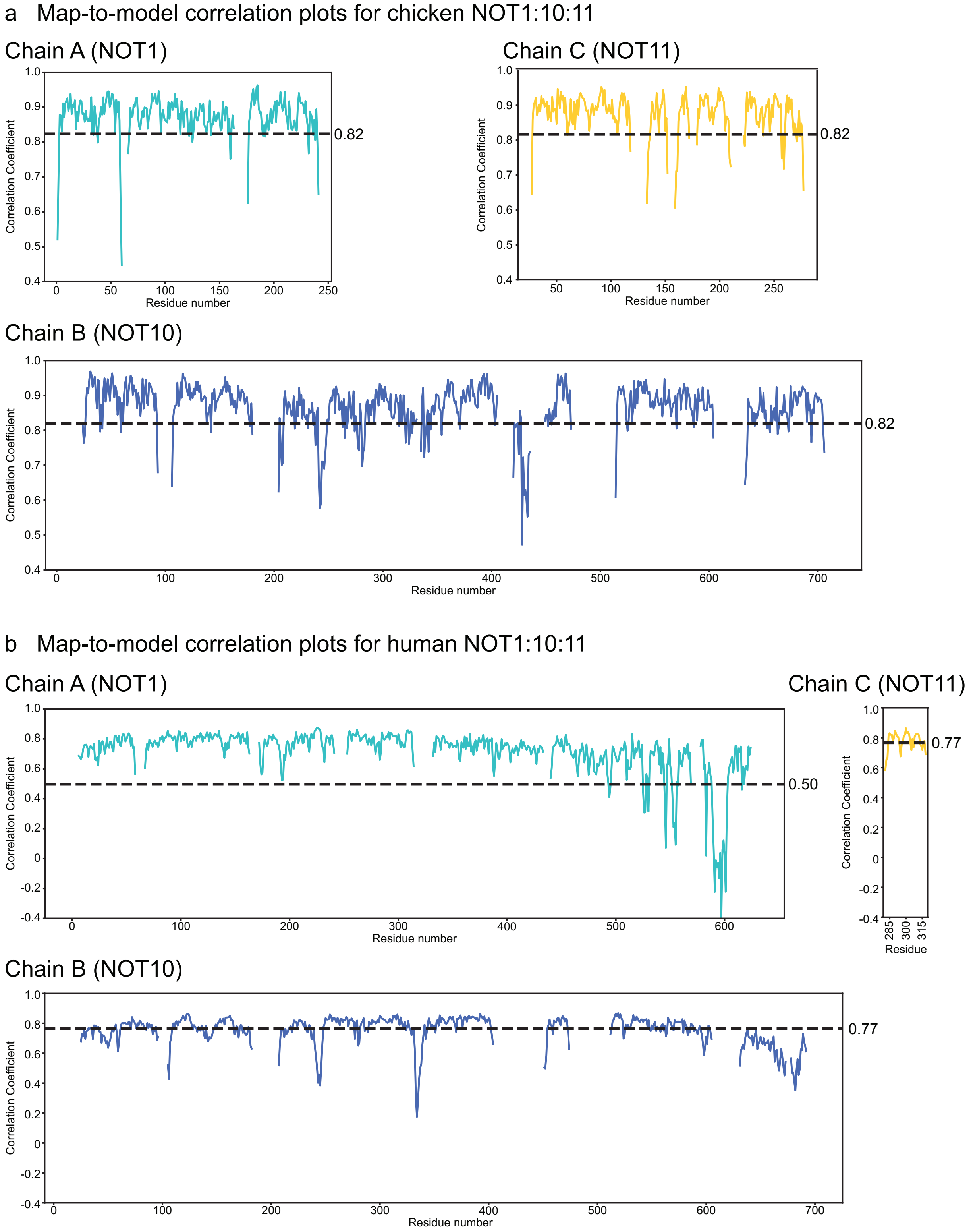

Supplementary Figure 6. Cryo-EM model-to-map correlations of NOT1:10:11

a) Map-to-model correlations for the cryo-EM structure of the chicken NOT1:10:11 complex.

b) Map-to-model correlations for the cryo-EM structure of the human NOT1:10:11 complex.

a Source data of Figure 3b

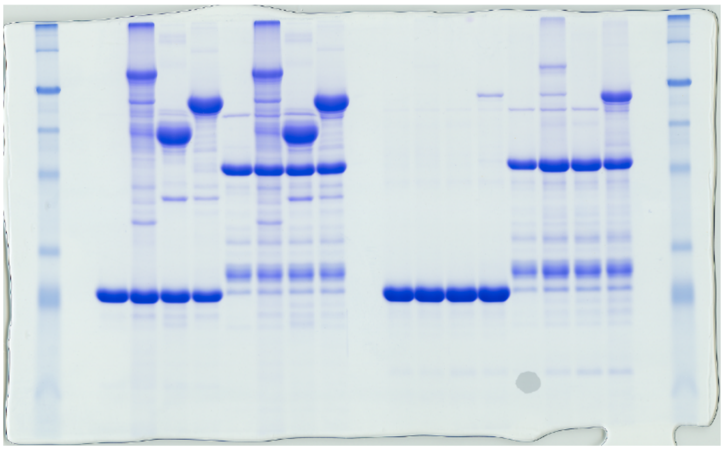

b Source data of Figure 3c

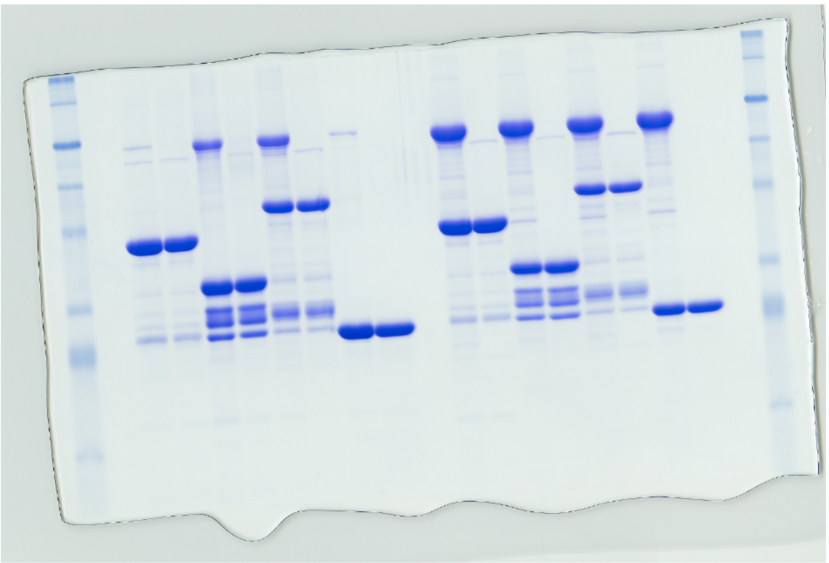

c Source data of Figure 3d

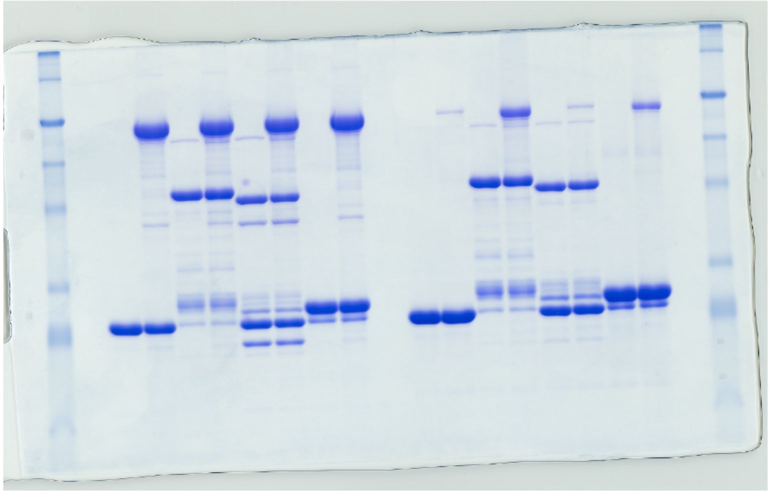

Supplementary Figure 7. Source Data of Fig. 3 b-d.

a) Uncropped SDS gel shown corresponding to Fig. 3b.

b) Uncropped SDS gel shown corresponding to Fig. 3c.

c) Uncropped SDS gel shown corresponding to Fig. 3d.

a Source data of Figure 3e

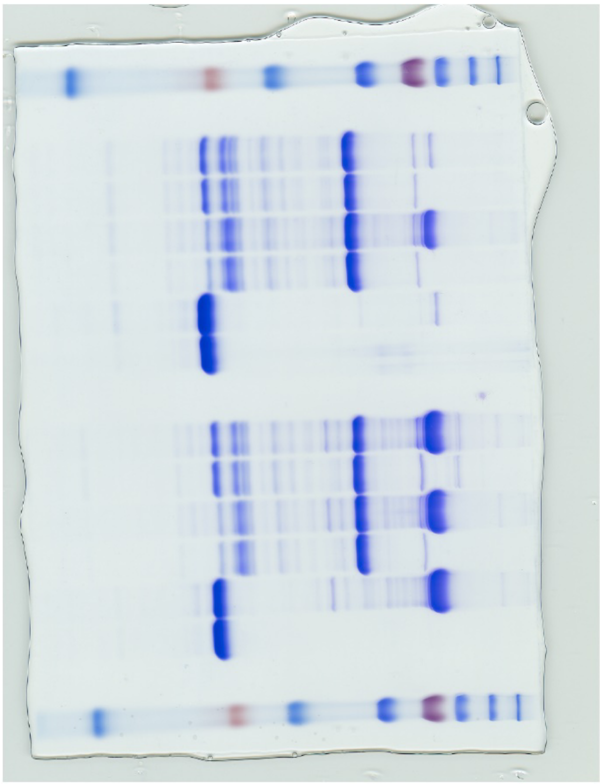

b Source data of Figure 3f

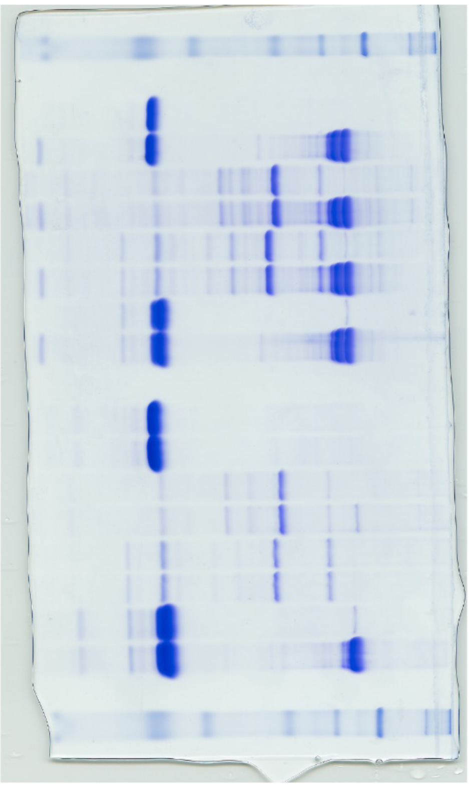

c Source data of Figure 3g

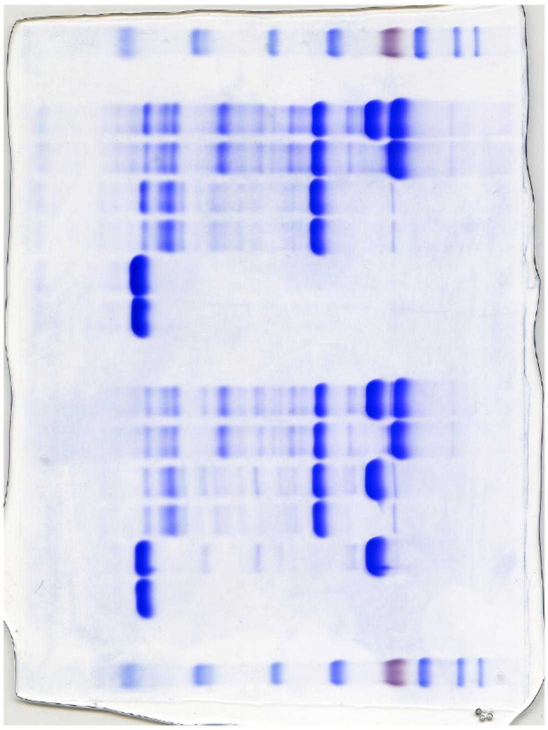

d Source data of Figure 3h

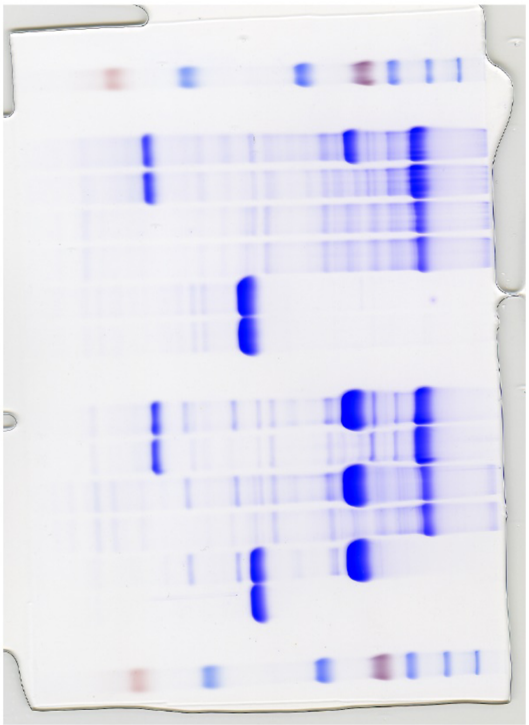

Supplementary Figure 8. Source Data of Fig. 3 e-h.

a) Uncropped SDS gel shown corresponding to Fig. 3e.

b) Uncropped SDS gel shown corresponding to Fig. 3f.

c) Uncropped SDS gel shown corresponding to Fig. 3g.

d) Uncropped SDS gel shown corresponding to Fig. 3h.
