# Supplementary material for: Structure and assembly of the NOT10:11 module of the CCR4-NOT complex
Source: Commun Biol. 2023 Jul 17;6:739. doi: 10.1038/s42003-023-05122-4 (PMC10352241; doi:10.1038/s42003-023-05122-4)
Supplement: Supplementary file 3 — Description of Additional Supplementary Files [file 42003_2023_5122_MOESM3_ESM.pdf]

Description of Additional Supplementary Files

File name: Supplementary Movie 1
Description: Overview of Cryo-EM reconstruction and model quality of the human NOT1:10:11 complex and map quality along the NOT11L.

File name: Supplementary Movie 2
Description: Overview of Cryo-EM reconstruction and model quality of the chicken NOT1:10:11 complex and map quality along the NOT11.
