# Supplementary material for: Structure and assembly of the NOT10:11 module of the CCR4-NOT complex
Source: Commun Biol. 2023 Jul 17;6:739. doi: 10.1038/s42003-023-05122-4 (PMC10352241; doi:10.1038/s42003-023-05122-4)
Supplement: Supplementary file 1 — Peer Review File [file 42003_2023_5122_MOESM1_ESM.pdf]

nature portfolio

Peer Review File

Structure and assembly of the NOT10:11 module of the CCR4-NOT complex.

Open Access This file is licensed under a Creative Commons Attribution 4.0 International License, which permits use, sharing, adaptation, distribution and reproduction in any medium or format, as long as you give appropriate credit to the original author(s) and the source, provide a link to the Creative Commons license, and indicate if changes were made. In the cases where the authors are anonymous, such as is the case for the reports of anonymous peer reviewers, author attribution should be to 'Anonymous Referee' followed by a clear attribution to the source work. The images or other third party material in this file are included in the article's Creative Commons license, unless indicated otherwise in a credit line to the material. If material is not included in the article's Creative Commons license and your intended use is not permitted by statutory regulation or exceeds the permitted use, you will need to obtain permission directly from the copyright holder. To view a copy of this license, visit http://creativecommons.org/licenses/by/4.0/.

This manuscript has been previously reviewed at another Nature Portfolio journal. This document only contains reviewer comments and rebuttal letters for versions considered at Communications Biology.

REVIEWERS' COMMENTS:

Reviewer #1 (Remarks to the Author):

The authors have provided sufficient responses to my earlier comments. It would be helpful to present more details on how the plots in the new Fig. S6 are generated. Is the correlation based only on the main-chain (or Ca) atoms? While deepEMhancer produced improvements in the EM reconstruction, the density for the side chains appears to be still rather weak for human NOT1 HEATc (Fig. 1c).

More detailed information on the comparison of the cryo-EM and crystal structures would be helpful. What are the rmsd values aligning the individual subunits/domains of the two structures? Also, Not11 does not seem to be included in Fig. S1b.

A point-by-point response to reviewer #1

The authors have provided sufficient responses to my earlier comments. It would be helpful to present more details on how the plots in the new Fig. S6 are generated. Is the correlation based only on the main-chain (or Ca) atoms? While deepEMhancer produced improvements in the EM reconstruction, the density for the side chains appears to be still rather weak for human NOT1 HEATc (Fig. 1c).

We thank the reviewer for the overall very positive and constructive interaction. The plots we report in Supplementary Fig. 6 have been calculated using the "Cryo-EM Validation tools" in Phenix. These tools report different overall correlation coefficients for the model vs. the reconstructed EM volume, i.e. the whole map, the main chain, the side chains and others. These values do not differ substantially, e.g. the six reported CCs for the human NOT1:10:11 complex range between 0.74 and 0.77. In addition, Phenix calculates the per-residue correlation coefficient plots which we report in Figure S6, which take into account all the modelled atoms, i.e. main chain and side chain atoms.

More info on this validation can be found on the Phenix website (https://phenix-online.org/documentation/reference/validation_cryo_em.html#model-and-data-fit) and in the original methods paper (https://doi.org/10.1107/S2059798318009324).

Indeed, as the reviewer points out, the quality of the reconstruction is relatively low for the human NOT1-HEAT$_C$ domain. The reason for this is the described flexibility of this domain, reflected by the complete disorder of the equivalent domain in the chicken structure and the relatively poor local Model-Map correlation coefficients in this region (Figure S6).

More detailed information on the comparison of the cryo-EM and crystal structures would be helpful. What are the rmsd values aligning the individual subunits/domains of the two structures? Also, Not11 does not seem to be included in Fig. S1b.

The reported rmsd values correspond to global rmsd values calculated using the "align" command in Pymol with the default cutoff value and only taking into account the Cα atoms.

For the individual domains, the rmsd values are as follows:

**Domain**	**Crystal vs. human Cryo-EM**	**Crystal vs. Chicken Cryo-EM**
NOT1 (all domains)	3.642 (485 Cα atoms)	0.461 (199 Cα atoms)
NOT1-HEAT$_N$	0.501 (178 Cα atoms)	0.446 (190 Cα atoms)
NOT1-HEAT$_C$	1.052 (238 Cα atoms)	*N/A (disordered)*
NOT10 (all domains)	0.819 (447 Cα atoms)	0.771 (482 Cα atoms)
NOT11 (all domains)	0.529 (37 Cα atoms)	1.061 (195 Cα atoms)
NOT11$_N$	*N/A (not part of the construct)*	0.604 (154 Cα atoms)
NOT11$_{Linker}$	0.529 (37 Cα atoms)	0.575 (39 Cα atoms)
NOT11$_{MIF4G}$	*N/A (disordered)*	*N/A (disordered)*
Complete structure	**1.392 (831 Cα atoms)**	**0.922 A (838 Cα atoms)**

NOT11 has indeed been omitted from Supplementary Fig. 1b by accident and we thank the reviewer for pointing this out. We have now prepared a new version of the figure that shows the NOT11$_{Linker}$ (the NOT11$_N$ and NOT11$_{MIF4G}$ domains present in the crystal structure have been omitted for clarity).
